# Epidemiological and genetic investigation of a cluster of cases of severe fever with thrombocytopenia syndrome bunyavirus

**DOI:** 10.1186/s12879-020-05072-w

**Published:** 2020-05-14

**Authors:** Lingling Mao, Baocheng Deng, Yuhong Liang, Yun Liu, Zijiang Wang, Jie Zhang, Wei Wu, Lei Yu, Wenqing Yao

**Affiliations:** 1 Liaoning Province Center for Disease Control and Prevention, Shenyang, Liaoning Province China; 2 Department of Infectious Diseases, The First Affiliated Hospital, China Medical University, Shenyang, Liaoning Province China; 3 Dalian Center for Disease Control and Prevention, Dalian, Liaoning Province China

**Keywords:** SFTS, Cluster outbreak, Molecular epidemiology, SFTSV

## Abstract

**Background:**

To analyze and discuss the transmission route of a cluster of cases of severe fever with thrombocytopenia syndrome bunyavirus (SFTSV).

**Method:**

We performed an epidemiological investigation and a genetic analysis of patients with severe fever with thrombocytopenia syndrome (SFTS) caused by SFTSV, their close contacts and the surrounding population.

**Results:**

We found that all patients had contact with the blood of the first patient. The comparison of gene sequences in the three isolated SFTSV strains showed that the strains were closely related. Six close contacts and nine individuals in the surrounding population were positive for SFTSV IgM antibody.

**Conclusion:**

We suspect that the cluster outbreak was transmitted via blood and that the natural reservoir host of SFTSV exists in the patients’ environment.

## Background

Severe fever with thrombocytopenia syndrome (SFTS) is a disease clinically characterized by fever, leukopenia, thrombocytopenia and multiple organ damage. It was first reported in the central and eastern regions of China in 2009, and the earliest cases can be traced back to 1996 [1, 2]. SFTS virus (SFTSV) was identified as the causative pathogen. SFTSV is classified into the Phlebovirus genus of the Phenuiviridae family. The genome of SFTSV is a single-stranded negativesense RNA and comprises three segments (S, M, L). The S segment contains 1744 nucleotides, the M segment contains 3378 nucleotides, and the L segment contains 6368 nucleotides [3]. SFTS is transmitted by tick bite s and contact with the blood or bodily fluid of SFTS patients [4, 5]. The average case-fatality rate of SFTS is 12% but can reach as high as 30% [6]. SFTS has become a serious threat to public health due to its high mortality and person-to-person transmission. SFTS was listed as one of the nine infectious diseases on the WHO priority list in 2017. In August 2014, an aggregation of SFTS cases was reported in a city in the southern area of Liaoning Province. We investigated these cases and used molecular epidemiological methods to study the epidemiologic features of the emerging infectious disease and confirm the infection source.

## Methods

### Subjects

The study subjects were the index patient, the close contacts of the index patient, and the members of the surrounding population of the city in the southern area of Liaoning Province where the SFTSV outbreak occurred in August 2014. There were four laboratory-confirmed SFTSV cases, two of which resulted in death. The diagnostic criteria were those recommended in the guideline for the prevention and treatment of severe fever with thrombocytopenia syndrome (2012 version) published by the Ministry of Health, PRC [7]. We identified six close-contact patients and fifty-five fellow villagers from the surrounding population.

### Epidemiological investigation

We performed an epidemiological case study of the four confirmed infected patients to study the potential transmission route of the family aggregation outbreak. To acquire the transmission mode among close contacts and the first infected patient, we collected 5 ml of venous blood from close contacts and individuals from the surrounding population. Additionally, sera from two cattle in the patient’s home and sera from two cattle in the neighbor’s home were also collected.

### Laboratory detection

#### Virus isolation and culture

We inoculated four blood samples into cultured Vero-E6 cells and changed the maintenance medium after 2 h at 37 °C. We observed the cytopathic effect (CPE) daily and collected virus from positive isolates when the CPE was above +++. The isolates were considered negative if no CPE appeared after blind passage for three generations [8]. Real-time fluorescence quantitative PCR (SFTSV qPCR Kit, DAAN Gene) was used to detect the collected virus.

#### Antibody detection in close contacts and the surrounding population

Blood samples from close contacts and individuals from the surrounding population were tested for immunoglobulin M (IgM) antibody to SFTSV by ELISA. The cattle blood samples were tested for immunoglobulin G (IgG) antibody to SFTSV by ELISA.

#### Sequencing and analysis of the complete genome of SFTSV

Nucleotides were extracted with EZ1 Advanced XL (QIAGEN). Twenty-two pairs of sequencing primers, three pairs for S fragments, seven pairs for M fragments and twelve pairs for L fragments, were designed, synthesized and provided by the Institute for Viral Disease Control and Prevention, Chinese Center for Disease Control and Prevention (CDC) [9]. The reaction system was prepared according to the protocol of the One-Step RT-PCR Kit (Promega). The reaction conditions were 60 °C for 1 min; 42 °C for 10 min; 50 °C for 30 min; 95 °C for 15 min; 35 cycles of 94 °C for 30 s, 55 °C for 30 s, and 72 °C for 1 min; and 72 °C for 10 min in a total volume of 50 μl. RT-PCR products were detected by agarose gel electrophoresis, and the positive products were two-way sequenced by Invitrogen-Life Technologies Corporation. Sequencing results were spliced by Contig Express, and sequence alignment and analysis were performed with sequences downloaded from GenBank by ClustalW and MEGA 4.1.

The nucleotides of the three SFTSV strains (LNDL2014–49 (O), LNDL2014–50 (O), LNDL2014–51 (O)) were extracted to amplify the L fragment (MT309099, MT309100, MT309101), M fragment (MT309102, MT309103, MT309104) and S fragment (MT309105, MT309106, MT309107), respectively. The target fragments were assembled to obtain the whole genome sequence of the three virus strains. We compared sequence similarity between our assembly sequences and eleven virus strains, six representative strains (Phlebovirus AH12/China/2010, Phlebovirus AH15/China/2010, Phlebovirus LN3/China/2010, Phlebovirus JS3/China/2010, Phlebovirus HN6/China/2010, and Phlebovirus SD24/China/2010) reported by the Chinese CDC in 2010 and five isolated strains from sporadic cases of SFTS in Liaoning Province in 2012. We constructed phylogenetic trees based on the results.

## Results

### Epidemiological investigation

The index patient was a peasant from a village in southern Liaoning Province, who presented fever (maximum of 39.7 °C), headache, and lethargy on July 28, 2014. The patient had a history of frequent farm work, but it was unclear whether he had been bitten by ticks. He received medical treatment at a local hospital but had no signs of remission. The patient presented severe leukopenia and thrombocytopenia upon admission to another hospital on August 5th. The patient was confirmed to have an SFTSV infection by the city Center for Disease Control and Prevention (CDC) on August 6th. The patient died at home after leaving the hospital on August 7th.

The second patient, a farmer with fever (maximum of 39.5 °C), lethargy, diarrhea and other symptoms, sought medical treatment on August 14, 2014 at a village clinic and county-level hospital but had no signs of recovery. He had no history of tick bites. The patient was found to have severe leukopenia and thrombocytopenia upon admission to an infectious disease hospital on August 21st. The patient was confirmed to have an SFTSV infection by the CDC and died on August 24th.

The third patient, presented fever (maximum of 39 °C), headache, nausea and other symptoms on August 17, 2014. He sought medical treatment at a village clinic, county-level hospital and an infectious disease hospital; he was found to have leukopenia and thrombocytopenia. The patient had no history of tick bites. The patient was confirmed to have an SFTSV infection by the CDC.

The fourth patient, a farmer and cousin of the index patient with fever (maximum of 39.5 °C), headache, vomiting, abdominal distension and other symptoms on August 18, 2014, sought medical treatment at a village clinic and county-level hospital. The patient had no history of tick bites. Laboratory testing showed leukopenia and thrombocytopenia. The patient was confirmed to have an SFTSV infection by the CDC on August 20th.

Due to the clustering of patients in this outbreak in the same village and all patients having typical clinical symptoms, including fever, thrombocytopenia, and leukopenia (Table 1), there was high suspicion of SFTS.

**Table 1:** Clinical features of a cluster of patients with severe fever with thrombocytopenia syndrome

	index patient	patient 2	patient 3	patient 4
Age range	60–70	70–80	50–60	60–70
Outcome	Death	Death	Discharge	Discharge
***Symptoms and signs***				
Maximal temperature, °C	39.7	39.5	39.0	39.5
Headache	Yes	Yes	Yes	Yes
Anergy	Yes	Yes	Yes	Yes
Nausea and vomiting	Yes	Yes	Yes	Yes
Bleeding	Yes	Yes	No	No
Diarrhea	Yes	Yes	Yes	No
Conjunctival congestion	Yes	Yes	No	No
Lymphadenopathy	Yes	Yes	No	No
Confusion	Yes	Yes	No	No
***Laboratory findings***				
White blood cell count, × 10^9^/L	1.5	1.77	1.25	1.3
Platelets count, ×10^9^/L	25.2	28	79	57
Proteinuria	Negative	Negative	Negative	Negative
Bleeding time	No data	Delayed	Normal	Normal
Coagulation time	Delayed	Delayed	Delayed	Normal
Blood SFTSV load (TCID50/ml)	4.69 × 10^7^	1.47 × 10^4^	4.41 × 10^2^	2.91
Virus isolation	Yes	Yes	Yes	No

**Fig. 1:**
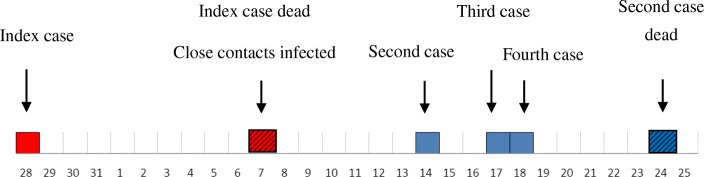
The onset time of cases

The index patient died at home on August 7th. Extensive blood was lost when the catheters were removed from the right chest of the patient. The third patient provided a haircut for the dead without gloves according to local custom. The second and the fourth patients contacted the blood of the index patient when they dressed the dead patient and moved the body (Fig. 1). The other six people, including the wife, sons, nephews and friends of the index patient, touched the body and blood of the index patient, but they had no signs of illness.

### Laboratory tests

#### Results of virus isolation and culture

The four positive samples confirmed by SFTSV nucleotide detection were inoculated into Vero-E6 cells. We found three positive samples by RT-PCR detection and collected the virus strains.

#### Antibody tests of close contacts

The results of testing the six close contacts were positive. Among the fifty-five fellow villagers, nine tested positive. The results of testing the patient’s cattle were positive, and the results of testing the neighbor’s cattle were negative.

#### Sequencing and analysis of the complete SFTSV genome

**Fig. 2:**
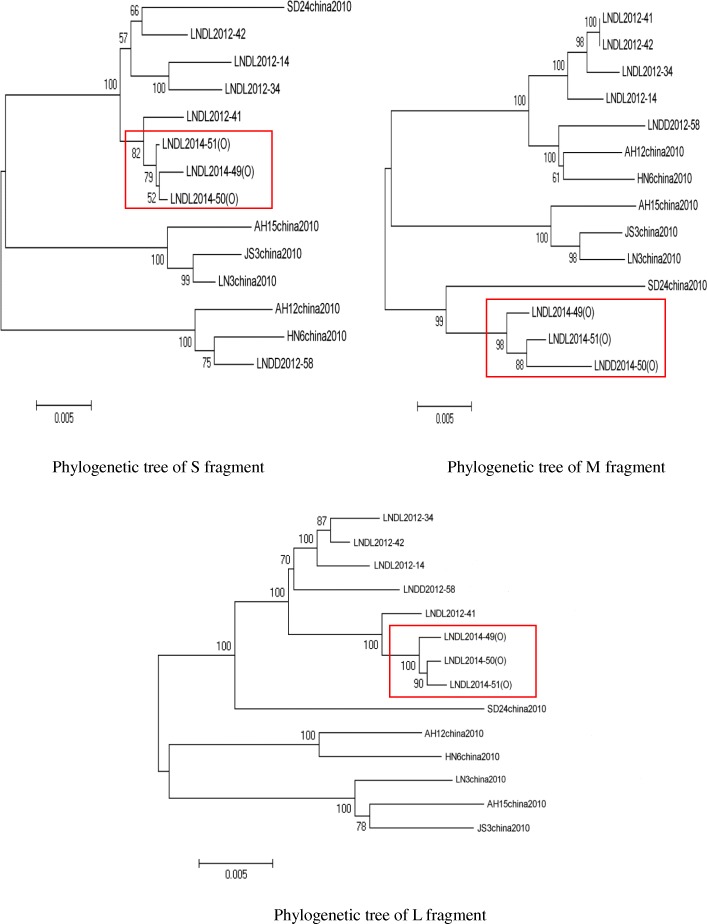
Whole genome phylogenetic tree of SFTSV

We compared sequence similarity between three SFTSV strains of outbreak and six representative strains reported by the Chinese CDC in 2010 and five isolated strains from sporadic cases of SFTS in Liaoning Province in 2012. The nucleotide sequences of the three SFTSV virus strains isolated from this outbreak were highly homologous, which indicated a close relationship among the isolated strains (Fig. 2). The nucleotide sequence identities of the three outbreak virus strains were 99.6 to 99.7% (L fragment), 98.8 to 99.4% (M fragment) and 99.7 to 99.8% (S fragment).

## Discussion

In recent years, human-to-human cluster outbreaks of SFTS have been reported in Central and East China [10–14]. This is the first epidemiological investigation report of a human-to-human cluster outbreak of SFTS in Liaoning Province.

In this aggregation epidemic, three secondary patients were infected by directly contacting the blood of the index patient when they cleaned and moved the body, cut the hair and changed the clothes of the dead without preventive measures. Although the six close contacts had no signs of illness, they were positive for IgM antibody. The results indicate that they were infected with SFTSV when they contacted the blood of the index patient. Based on the time when the secondary patients contacted the blood of the index patient and the onset time, we estimate that the incubation period for the human-to-human transmission of SFTSV is 7 ~ 11 days, which is similar to the result reported by Xu Zhuping [15]. Jimin Sun [16] reported that SFTSV infection and fatality may be associated with genetic susceptibility; this family cluster outbreak indicates such a possibility. We performed sequencing and analysis of the whole genome of three SFTSV virus strains isolated from this outbreak. The result indicates that these SFTSV virus strains were very closely related, confirming by molecular epidemiology methods that this outbreak is a human-to-human cluster outbreak. The results of the phylogenetic tree of the S, M and L fragments showed that the three SFTSV virus strains from this outbreak, strains from sporadic cases from a local area in 2012, and isolated strains from Shandong Province were in the same branch, which indicates that the SFTSV virus strains have a geographical distribution trend [17]. In the phylogenetic tree of the M fragment, there were slight differences among the three SFTSV virus strains from this outbreak and strains from sporadic cases from the local area in 2012, which indicates that the virus strains have some mutations on some loci.

There were nine positive results of SFTSV antibody detection in fellow villagers, and they had no relationship to this outbreak, as confirmed by epidemiological investigation. Additionally, positive results of SFTSV antibody were detected in the patient’s cattle. This phenomenon shows that a natural reservoir host of SFTSV exists in the environment of this village. We visited and confirmed that ticks were distributed around the village and that tick bites occurred frequently. Because the investigation was performed at the beginning of September, we failed to capture virus -carrying ticks. The situation of the natural reservoir host of SFTSV in this village should be further studied.

In this study, all patients were farmers and resided in the same village. We can’t rule out the possibility that the secondary patients acquired their infection from the environment or tick bites rather than from contact the first case. With regard to the serological positive of surrounding close contact, we only had one blood collection. We can’t compared with the titer of first results, where that of the second results from the sera collected from the same close contacts two or 3 weeks later increased or not. All of these are the limitations of this study.

## Conclusions

SFTS is an emerging infectious disease. Clinicians have little knowledge about this disease in some areas; therefore, it is easy for SFTS to evade diagnosis or be misdiagnosed. Some rural areas retain traditional funeral customs. When the patients were dying in the hospital, the family requested discharge from the hospital and handled the funeral in the village, which increased the risk of human-to-human infection and outbreak. Strengthening the monitoring and training at medical and health institutions; improving the recognition, diagnosis, treatment, reporting and handling capacity of cases of SFTS; strengthening public health education; and propagating prevention and control knowledge are all measures that have great significance for preventing the transmission of SFTS among humans.

## Data Availability

The SFTSV gene sequence has been uploaded Geenbank included: MT309099, MT309100, MT309101, MT309102, MT309103, MT309104, MT309105, MT309106, MT309107.

## Abbreviations

SFTS: Severe fever with thrombocytopenia syndrome
SFTSV: Severe fever with thrombocytopenia syndrome bunyavirus
CPE: Cytopathic effect
IgM: Immunoglobulin M
IgG: Immunoglobulin G
CDC: Chinese Center for Disease Control and Prevention
